# HIPrevision - a multicenter prospective randomized control superiority trial of antimicrobial IP-coated revision hip prostheses versus non-IP-coated comparators for the revision of periprosthetic hip joint infections

**DOI:** 10.1186/s13063-026-09541-6

**Published:** 2026-02-17

**Authors:** Volker Alt, Nike Walter, Francisco Baixauli, Jerzy Bialecki, Lutz Dreyer, Jens Goronzy, Sebastian Hardt, Dirk Herold, Daniel Kendoff, Stefan Maenz, Dominik Rak, Maximilian Rudert, Andrzej Sionek, Maik Stiehler, Sebastian Meller

**Affiliations:** 1 Klinik und Poliklinik für Unfallchirurgie, Universitätsklinikum Regensburg, Regensburg, 93053 Germany; 2 Servicio de Cirugia ortopédica y traumatologia, Hospital Universitario y Politécnico la Fe, Valencia, Spain; 3 Gruca Orthopedic and Trauma Teaching Hospital CMKP, Otwock, Poland; 4 Aesculap AG, Tuttlingen, Germany; 5 UniversitätsCentrum für Orthopädie, Unfall- & Plastische Chirurgie, Universitätsklinikum Carl Gustav Carus, Technischen Universität Dresden, Dresden, Germany; 6 Centrum für Muskuloskeletale Chirurgie (CMSC), Charité Universitätsmedizin Berlin, Campus Charité Mitte, Berlin, Germany; 7 Klinik für Orthopädie und Unfallchirurgie, Caritas-Krankenhaus Bad Mergentheim, Bad Mergentheim, Germany; 8 Zentrum für Orthopädie und Unfallchirurgie, Helios Klinikum Berlin-Buch, Berlin, Germany; 9 Orthopädische Klinik König-Ludwig-Haus, Universität Würzburg, Würzburg, Germany; 10 Berlin Institute of Health, Center for Musculoskeletal Surgery (CMSC), Charité-Universitätsmedizin Berlin, Corporate Member of Freie Universität Berlin, Humboldt-Universität zu Berlin, Berlin, Germany

**Keywords:** Hip, Silver, Coating, Periprosthetic joint infection, PJI, Hip revision arthroplasty, Re-revision

## Abstract

**Background:**

Periprosthetic joint infection (PJI) is one of the most severe complications in hip arthroplasty, and treatment of PJI is associated with high re-infection rates and significant loss of quality of life for patients, as well as socio-economic impact to health systems. Antimicrobial coating of implants with silver is a promising option to improve the outcome of PJI. However, there is no data from randomized control trials on silver-coated versus standard non-silver-coated implants in patients with PJI. Therefore, the aim of the current study is to assess clinical outcome for the use of silver-coated hip implants (further referred to as IP-coated) versus non-IP-coated implants in patients requiring surgical revision for hip PJI.

**Methods:**

This is a multicenter confirmatory interventional randomized controlled superiority single-blinded study with two stages: pilot stage (part A) and pivotal stage (part B). Patients indicated for unilateral cementless acetabular and hip stem revision due to chronic periprosthetic infection planned for single-stage or two-stage surgical procedures are included. Patients will randomly be assigned either to the IP-coated or the non-IP-coated implant group. The primary outcome parameter is infection-free survival within 12 months after hip PJI. The underlying hypothesis is that the IP coating significantly reduces the risk of periprosthetic reinfection compared to the non-IP-coated implants. Secondary outcome parameters include data on the safety and performance of the prostheses through a 2-year clinical follow-up, including patient-related outcome parameters, such as Harris Hip Score, EQ-5D, radiographical assessment, and blood silver concentrations. An adaptive study design is planned with the inclusion of 268 subjects according to initial sample size calculations. Upon follow-up of 134 patients for 12 months or inclusion of 90% of the study patients, preliminary study results will be used to re-estimate the sample size, which may be extended up to 400 subjects.

**Discussion:**

The current study aims to fill the evidence gap for the idea of IP-coated implants to reduce re-infection rates in the treatment of hip PJI. To the authors’ best knowledge, this is the first randomized controlled superiority trial evaluating the outcome of a silver-coated versus a non-silver-coated prosthesis for the treatment of hip PJI. The adaptive study design allows an adjustment of the initial sample size to reduce the risk of an underpowered superiority.

**Trial registration:**

ClinicalTrials.gov NCT06737809 (date of publication 17.12.2024).

## Administrative information

**Table:** 

Title {1}	HIPrevision — a multicenter prospective randomized control superiority trial of antimicrobial IP-coated revision hip prostheses versus uncoated comparators for the revision of periprosthetic hip joint infections
Trial registration {2a and 2b}	ClinicalTrials.gov identification: NCT06737809 Eudamed identification: CIV-24-05-047447
Protocol version {3}	2025 March 21 Version 5.0
Funding {4}	This study is sponsored by Aesculap AG (sponsor’s study identification: AAG-G-H-2023)
Author details {5a}	^1^Klinik und Poliklinik für Unfallchirurgie, Universitätsklinikum Regensburg, Regensburg, 93053, GermanyKlinik und Poliklinik für Unfallchirurgie, Universitätsklinikum Regensburg, Regensburg, 93053, Germany. ^2^Servicio de Cirugia ortopédica y traumatologia, Hospital Universitario y Politécnico la Fe, Valencia, Spain. ^3^Gruca Orthopedic and Trauma Teaching Hospital CMKP, Otwock, Poland. ^4^Aesculap AG, Tuttlingen, Germany. ^5^UniversitätsCentrum für Orthopädie, Unfall- & Plastische Chirurgie, Universitätsklinikum Carl Gustav Carus,Technischen Universität Dresden, Dresden, Germany. ^6^Centrum für Muskuloskeletale Chirurgie (CMSC), Charité Universitätsmedizin Berlin, Campus Charité Mitte, Berlin, Germany. ^7^Klinik für Orthopädie und Unfallchirurgie, Caritas-Krankenhaus Bad Mergentheim, Bad Mergentheim, Germany. ^8^Zentrum für Orthopädie und Unfallchirurgie, Helios Klinikum Berlin-Buch, Berlin, Germany. ^9^Orthopädische Klinik König-Ludwig-Haus, Universität Würzburg, Würzburg, Germany. ^10^Berlin Institute of Health, Center for Musculoskeletal Surgery (CMSC), Charité-Universitätsmedizin Berlin, Corporate Member of Freie Universität Berlin, Humboldt-Universität zu Berlin, Berlin, Germany.
Name and contact information for the trial sponsor {5b}	Aesculap AG, Am Aesculap Platz, 78532 Tuttlingen, Germany Aesculap AG is part of the B. Braun Group
Role of sponsor {5c}	The sponsor is the manufacturer of the investigational device

## Introduction

### Background and rationale {6a}

Total hip arthroplasty (THA) is one of the most frequently performed procedures in orthopedic surgery, offering significant pain relief, functional restoration, and improved quality of life for patients. Annually, over 3.1 million primary THAs are performed across Europe, with 177,826 procedures recorded in Germany alone in 2023 [1]. However, periprosthetic joint infection (PJI) remains one of the most severe complications following THA, placing a heavy burden on both patients and healthcare systems [2–5]. From prospective or registry studies, periprosthetic infection rates (or if not explicitly stated, infection-related re-revision rates) of 3.7%, 12%, 12.5%, 15.9%, 18.2%, 18.6%, 22.0%, and 23.3% have been reported after infection-related hip revision arthroplasty. Registry analyses suggest that 1-year revision rates following multiple revision surgeries may be significantly higher-by at least 49%-compared to rates after first-time revision procedures.

To address this clinical challenge, an infection prevention (IP) silver coating (further referred to as IP coating, Aesculap, Tuttlingen, Germany) will be used for hip revision prosthesis implants (Plasmafit^®^ Revision cup, Structan^®^ Augments, and Prevision^®^ Revision stem system, Aesculap, Tuttlingen, Germany) as infection prevention and for the surgical treatment of hip PJI. This novel coating developed by Bio-Gate AG, Nürnberg, Germany, is based on metallic silver embedded in a polysiloxane matrix, designed to release antimicrobial silver ions upon water contact [6, 7]. This IP coating relies on the multimodal effects of released silver ions, such as alterations to the bacterial cell respiratory system and DNA, with a broad coverage of all types of microorganisms, including gram-positive and gram-negative bacteria [8].

Preclinical and clinical data suggest that this approach can inhibit bacterial colonization at the implant surface without significantly affecting the surrounding tissue, offering a targeted strategy for the use of coating in PJI [9].

The used investigational IP-coated devices are similar to their non-IP-coated CE-certified predecessors in terms of mechanical properties and clinical indications. Clinical safety of the used silver ions in the coating has been shown recently [10]. Given the substantial burden of PJIs and the unmet clinical need for effective prevention strategies, evaluating the efficacy of IP-coated implants is both timely and clinically relevant. The proposed clinical investigation will assess whether this novel silver coating technology can significantly reduce PJI rates in PJI revision hip arthroplasty, ultimately improving patient outcomes.

### Objectives {7}

The aim of this clinical investigation is to evaluate the safety and performance of the IP-coated hip implants. The primary endpoint will assess the effectiveness of the IP-coated hip implants in reducing the risk of periprosthetic joint infection. Secondary endpoints will examine the key functional and clinical benefits of the implants, including hip joint functional restoration, radiological outcome, and pain reduction, alongside an evaluation of device safety. Non-IP-coated hip implants will be utilized as the comparator device in this study.

### Trial design {8}

This clinical investigation is a parallel-group, randomized, controlled trial with a 1:1 allocation ratio following a superiority framework. This clinical investigation consists of two stages: pilot stage (part A) and pivotal stage (part B).

The two stages of the clinical investigation allow an early assessment of short-term safety of the investigational products at the beginning (part A) with a low number of subjects. This is followed by the main clinical investigation (part B). In addition, an adaptive study design allows the extension of the sample size at interim analysis during part B.

## Methods: participants, interventions, and outcomes

### Study setting {9}

This study is carried out at 10 study sites in Poland, Spain, and Germany.

### Eligibility criteria {10}

#### Inclusion criteria

Participants eligible for inclusion in the study are male and female patients aged 18 years or older who have provided written informed consent prior to participation. Eligible participants must have been diagnosed with a periprosthetic joint infection (PJI) according to the European Bone and Joint Infection Society (EBJIS) criteria [11], with the infection classified as chronic based on the recommendations of the Pro-Implant Foundation (PIF, Berlin, Germany) [12]. These patients must be indicated for unilateral, cementless acetabular and hip stem revision surgery due to chronic periprosthetic infection, which may involve either a single-stage or two-stage surgical procedure, as determined by the clinical standards of the investigation site. Additionally, participants must be assessed by the clinical investigator as therapy compliant and capable of attending all follow-up visits required by the study protocol. Finally, only patients with an American Society of Anesthesiologists (ASA) physical status classification of I to III prior to explantation of the infected prosthesis will be included in the study [13].

#### Exclusion criteria

Patients meeting any of the following criteria will be excluded from the study: Simultaneous participation in another interventional clinical trial involving drugs or medical devices will result in exclusion. Additionally, patients for whom the investigational implant components (femoral and/or acetabular) are deemed unsuitable due to anatomical or bone defect considerations, including cases requiring additional plate osteosynthesis, will not be eligible.

Patients with a previously implanted silver-coated device will also be excluded, as will those with immunodeficiency, such as individuals receiving immunosuppressive therapy or those with severe immunodeficiency requiring treatment. However, rheumatoid arthritis is explicitly not considered a reason for exclusion. Furthermore, patients undergoing active cancer treatment, including chemotherapy or radiation therapy, will not be included.

Severe soft tissue defects requiring local or free flap procedures, as well as periprosthetic joint infections with evidence of fungal infection, represent further exclusion criteria. Patients on indefinite antibiotic suppression therapy at the time of screening, defined as continuous administration of antibiotics with a non-curative intent, are not eligible. Similarly, individuals with a body mass index (BMI) greater than 40 kg/m^2^ will be excluded.

Based on the patient’s self-reported information and investigator assessment, women who are pregnant or breastfeeding, or those of childbearing potential not using adequate contraception, are ineligible for participation. Patients with known or self-reported hypersensitivity to silver or titanium will also be excluded.

Additional exclusions apply to patients held in custodial settings or correctional facilities and those in a dependent relationship with the sponsor, clinical site, or investigator. Contraindications to the investigational or comparator devices will also preclude participation. These include secondary diseases impacting the function of the joint implant, severe osteoporosis or osteomalacia, poor bone quality or osseous malformations that compromise the stability of joint replacement anchorage, non-regenerative bone conditions with insufficient proximal femoral support, and cases requiring prosthesis heads with neck length XXL.

### Who will take informed consent? {26a}

Informed consent will be obtained by a qualified investigator or by trained study personnel delegated by the principal investigator before any study-related procedures are performed. The process will involve a comprehensive explanation of the study aims, procedures, risks, benefits, and the participant’s rights, ensuring adequate time for questions and voluntary decision-making.

### Additional consent provisions for collection and use of participant data and biological specimens {26b}

Participants will provide consent for the collection, processing, and storage of personal and health data in compliance with GDPR and applicable data protection laws. Biological specimens collected during the study will only be used for the purposes defined in the protocol and will not be stored or analyzed beyond the scope of this trial.

## Interventions

### Explanation for the choice of comparators {6b}

The comparator devices selected for this study are non-IP-coated acetabular revision cups, acetabular augments, and revision hip stems, which represent the current standard of care for cementless revision hip arthroplasty and are CE-marked (Plasmafit^®^ Revision cup, Structan^®^ Augment, and Prevision^®^ Revision stem system, Aesculap, Tuttlingen, Germany). These implants are widely used in clinical practice and have established safety and performance profiles for hip arthroplasty revision.

Patients in the investigational group will receive IP-coated cementless acetabular cups, augments, and hip stems (Plasmafit^®^ Revision cup, Structan^®^ Augments, and Prevision^®^ Revision stem) during their revision hip arthroplasty procedure. Patients in the comparator group will receive non-IP-coated implants that are otherwise identical in design and mechanical properties to the investigational devices. All implants will be used in accordance with the manufacturer’s surgical manual and clinical guidelines. The surgical procedure will follow standard practices for revision hip arthroplasty, including preoperative planning, intraoperative implant positioning, and fixation. Patients can only be included if both the acetabular and femoral prostheses are exchanged, and the investigational devices are suitable for the revision procedure. Articulation options, including a modular dual mobility option, may be chosen according to patient-specific and surgical factors. The selection of implant sizes and components will be based on preoperative imaging and intraoperative assessment of patient anatomy and bone defects.

### Intervention description {11a}

The intervention is the implantation of a permanent implant. A revision will only be performed if necessary due to medical reasons. Both investigational and comparator devices will be implanted during a single surgical procedure performed as part of a one-stage or two-stage revision hip arthroplasty, depending on the clinical indication and surgeon’s preference. All patients will undergo the same preoperative preparation, intraoperative protocols, and postoperative follow-up procedures, ensuring consistency across the two groups. Follow-up visits will be conducted according to the study protocol, with data collected on infection rates, functional and radiological outcomes, pain levels, and safety parameters at predefined time points.

### Criteria for discontinuing or modifying allocated interventions {11b}

Discontinuation or modification of the assigned intervention will be considered in cases of serious adverse events, patient withdrawal of consent, or if continuing the assigned treatment is deemed medically inappropriate by the investigator. Any such decisions will be thoroughly documented, and alternative standard treatments will be provided as clinically indicated.

### Strategies to improve adherence to interventions {11c}

Adherence to the intervention protocol will be facilitated through comprehensive training of surgical and study staff, regular monitoring visits, and detailed documentation of all procedural steps in electronic case report forms (eCRFs). Any deviations from the protocol will be recorded and addressed promptly.

### Relevant concomitant care permitted or prohibited during the trial {11d}

Participants will receive concomitant care and medications based on clinical standards applicable to the treatment of PJI. No specific prohibitions on concurrent therapies unrelated to infection management exist.

Following completion of the trial, participants will be treated according to the standard of care practice of the treating surgeon.

### Provisions for posttrial care {30}

The sponsor will maintain an adequate insurance policy covering damages arising out of the clinical trial. This insurance covers the subjects with respect to the risks involved in this study according to the clinical investigational plan.

### Outcomes {12}

The primary endpoint is the infection-free survival rate, evaluated to demonstrate that revision hip implants with the antimicrobial silver coating significantly reduce the rate of periprosthetic reinfections within 12 months after hip revision arthroplasty. The primary variable is the occurrence of periprosthetic joint infection classified as “infection likely” or “infection confirmed” according to the European Bone and Joint Infection Society (EBJIS) criteria [11]. The time point for assessing this outcome is 12 months postoperatively, as the silver coating is designed to provide efficacy within this period. 

Secondary endpoints will be functional outcomes assessed using the Harris Hip Score (HHS) [14], focusing on pre- and postoperative scores to determine improvements in hip functionality, as well as quality of life evaluated through the EQ-5D-5L questionnaire [15]. Further, Kaplan-Meier implant survival will be determined for endpoints including septic revisions and all-cause revisions. Radiological outcome (hip stem migration, radiological signs of loosening, radiologic observations in the fixation area) will be evaluated using standardized X-rays. Safety outcomes including device failure, signs of systemic or local argyria, and (S)AEs and (S)ADEs will be documented. In addition, specific laboratory parameters related to liver (ALT, AST, GGT) and kidney (eGFR) function, as well as inflammatory markers (e.g., CRP, leukocytes), silver ion in peripheral blood and drainage liquid, and surgery-related data, including bone defect classification, length of stay, and antibiotic therapy details will be evaluated.

### Participant timeline {13}

**Fig. 1:**
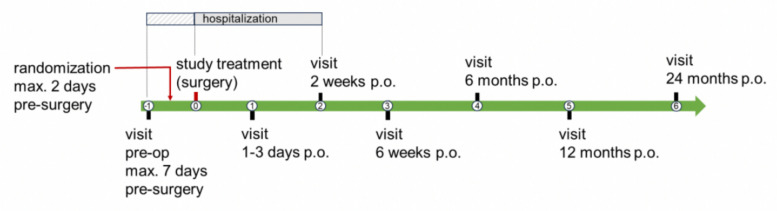
Planned study visits

The clinical investigation covers eight study-related visits (Fig. 1): one preoperative visit, the surgery itself (implantation of the investigational or comparator device), and six postoperative (p.o.) visits. All visits are within the frame of clinical standard visits. An overview of the schedule of enrollment, interventions, and assessments is shown in Table 1.

**Table 1:** Participant timeline: schedule of enrollment, interventions, and assessments

		Post-randomization			Follow-Up			
Timepoint	*−7 to −2*	0	*1 to 3 days*	*2 weeks*	*6 weeks*	*6 months*	*12 months*	*24 months*
Enrollment								
Eligibility screen	X							
Informed consent	X							
Randomization	X							
Intervention/comparator								
*IP-coated device*		X						
*Non-IP-coated device*		X						
Assessments								
*Infection-free implant survival rate (signs of required revision)*			X	X	X	X	X	X
*Harris-Hip-Score, Eq-5D-5L*	X		X	X	X	X	X	X
*Radiological outcome (hip stem migration, signs of loosening, fixation) *via* X-rays*		X		X			X	X
*Safety (laboratory blood parameter, signs of systemic or local argyria, (S)AEs/(S)ADE, device failure)*			X	X	X	X	X	X

### Sample size {14}

The sample size calculation for this study is based on the 1-year event rate of PJI in the comparator group, assumed to be 21.5%. This assumption is derived from a custom analysis of the EPRD (German Arthroplasty Registry) data [1], which documents revision procedures where implant components are exchanged, including Kaplan–Meier estimates for re-revision rates (total, infection related, and non-infection related) at 6, 12, and 24 months. Specifically, the re-revision rate due to periprosthetic infection within 12 months, for procedures involving combined exchange of stem and cup, was reported as 16.4%. However, this figure only accounts for cases where reinfections resulted in implant exchange and does not fully capture all events classified under the EBJIS criteria (“infection likely” or “infection confirmed”). To address this, a correction factor of 1.33 was applied to account for cases of reinfection treated with alternative approaches, such as antibiotic suppression therapy, implant retention surgery (DAIR), amputation, or where infection was not documented at the time of registry entry. After this adjustment, the estimated event rate in the comparator group increased to 21.5%.

In the intervention group, the event rate is assumed to be 8.6%, reflecting an anticipated 60% reduction in the rate of periprosthetic reinfections. The effect size estimation is based on the estimated reduction of periprosthetic infections in humans by 90% (unpublished case series), a reduction of periprosthetic infections in a rabbit model by 65% [16], and a reduction of infections in canine osteosynthesis implants by 73% (unpublished manufacturer data) or by 39% in the same canine implant type based on a different study [17].

Using a one-sided chi-square test for independent two-sample proportions with a significance level of *α* = 0.025 and a power of 80%, the study requires 240 participants (120 per group) to meet the primary endpoint. To account for a 10% drop-out rate, a total of 268 patients will be enrolled, with 20 patients allocated to part A and 248 patients to part B. Sample size was calculated using SAS (SAS VIYA software version V.04.00, SAS Institute Inc., Cary, NC, USA).

The above chi-squared based sample size calculation was used, although log-rank tests are used for primary hypothesis tests. A comprehensive simulation-based sample size calculation using the log-rank statistics under variation of the underlying hazard function found a small to marginal increase in power compared to the chi-squared statistic.

As predefined in the protocol, an extension of the total sample size of up to 50% is allowed, which results in a maximum of 400 patients (360 patients + 10% dropout). The 50% limit was determined based on practical considerations. This results in 20 patients scheduled for the safety phase (part A) and up to 380 patients scheduled for the main phase (part B).

The sample size re-estimation will be based on all available data of the first 134 treated patients, limited to their 12 months follow-up visit (“right truncated”). By estimating the per-group hazard, based on the corresponding survival data, the conditional power for detecting the difference in primary outcome between the two groups in the final analysis will be re-estimated. Details can be found in Wassmer and Brannath [18]. In case the intended number of 268 study subjects yields a conditional power smaller than 80% for the log-rank test in the final primary analysis, the number of subjects to be included in the study is increased up to the prespecified limit of 400 patients. When the conditional power based on the initially planned 268 patients is 80% or above, the clinical investigation will continue using the planned sample size. If the conditional power is very small (e.g. < 10%), the study will be completed with the originally estimated sample size.

### Recruitment {15}

Recruitment will be supported by providing study site investigators with clear enrollment targets, regular performance feedback, and educational materials for potential participants. Engagement activities such as investigator meetings and outreach within clinical networks will promote awareness and enrollment adequacy.

## Assignment of interventions: allocation

### Sequence generation {16a}

Participants will be randomized in a 1:1 ratio with random block sizes to either the investigational or comparator group, with site-specific randomization lists to ensure balanced allocation.

### Concealment mechanism {16b}

Randomization will be implemented using a secure, validated electronic data capture system with concealed allocation sequences. The randomization code will not be accessible to investigators until allocation, preventing selection bias.

### Implementation {16c}

The randomization will be implemented via the electronic data capture system secuTrial^®^ system to ensure allocation concealment. The system restricts access to the allocation sequence until the point of randomization, ensuring that the investigator is not aware of group assignment in advance. Randomization of eligible patients will be requested by the investigator after preoperative examination and timely before the planned date of surgery.

## Assignment of interventions: blinding

### Who will be blinded {17a}

The participants will be blinded to the intervention group they are allocated to, while the surgeons performing the procedures cannot be blinded due to visible differences in the investigational and comparator devices. Radiological analyses will also be conducted in a blinded manner through central radiology analysis.

### Procedure for unblinding if needed {17b}

The study is designed to remain blinded for the participants until the closure of the database and determination of the analysis populations.

## Data collection and management

### Plans for assessment and collection of outcomes {18a}

The assessment and collection of outcomes in this clinical investigation will follow a structured approach to ensure data quality and reliability. Data will be collected using electronic case report forms (eCRFs), ensuring compliance with GDPR regulations.

### Plans to promote participant retention and complete follow-up {18b}

Retention will be promoted by flexible scheduling of follow-up visits, appointment reminders via telephone or electronic communication, and providing participants with direct contact points for study-related support or questions. Missed visits will be promptly rescheduled to minimize data loss.

### Data management {19}

When a patient agrees to participate in the clinical investigation and has signed the informed consent form, the study team at the investigation site will allocate a unique code, the subject identification (ID), to the patient. Relevant personal data, health data, and procedural information necessary to conduct the clinical investigation will be collected under this subject ID. Information to be reported to the sponsor is recorded in an eCRF. Data management including creation of eCRFs including data plausibility check, data validation, data cleaning and query procedure, medical coding, and handling of the database will be performed according to designated SOPs. A data management plan (DMP) was prepared before the start of the clinical investigation.

### Confidentiality {27}

All data will be collected in compliance with the General Data Protection Regulation (GDPR) and other applicable laws. Identifiable information is pseudonymized, with only the investigators having access to re-identification keys. Data shared with the sponsor or external parties is limited to de-identified datasets. Participants are informed of data handling, storage, and potential access by authorized personnel during the informed consent process.

### Plans for collection, laboratory evaluation, and storage of biological specimens for genetic or molecular analysis in this trial/future use {33}

The collected biological specimens will not be used for genetic or molecular analysis, nor are they intended for future ancillary studies beyond the scope of this trial.

## Statistical methods

### Statistical methods for primary and secondary outcomes {20a}

The primary endpoint of this study is the comparison of infection-free survival rates (periprosthetic infections) within 12 months after revision total hip arthroplasty (THA) due to periprosthetic joint infection. The following hypothesis will be tested:

- H₀ (null hypothesis): hT ≥ hC
- Hₐ (alternative hypothesis): hT < hC

where hT represents the hazard function in the treatment group and hC represents the hazard function in the control group for the 12-month postoperative period.

The primary null hypothesis is tested using a one-tailed inverse normal combination test at *α* = 0.025, allowing for a two-stage adaptive study design that combines log-rank tests for each stage. The null hypothesis is rejected if the combined *p* < 0.025. The *p*-values for each stage (*p*₁ and *p*₂) will be calculated using a one-sided log-rank test for periprosthetic reinfections (as defined by EBJIS criteria) within 12 months post-treatment. Kaplan–Meier survival curves will be right-censored at 12 months.

Descriptive statistics or comparative analyses between the treatment and control groups for demographic data, safety, and performance variables will be conducted where appropriate. Functional outcome and quality of life in the treatment groups between treatment arms will be evaluated using a linear regression model with repeated measures. Two-year implant survival will be evaluated according to the Kaplan-Meier method and compared in the treatment arms with log-rank test for the events periprosthetic joint infections, septic revisions, and all-cause revisions. Occurrence of any radiolucent line, and occurrence of hip stem migration > 2 mm will be analyzed by using a logistic regression model with repeated measures, as well as the mean migration in patients with relevant migration (> 2 mm) by using a linear regression model with repeated measures. Safety outcomes of adverse events, device failure, and component revision between treatment arms will be evaluated by using a logistic regression.

All analyses will be based on the eligible randomized patient population. A CONSORT diagram will be used to visualize the number of participants screened, randomized, treated, and analyzed for the primary outcome. Secondary analyses will be exploratory. Secondary analyses will be performed without confirmatory character:

- Functional outcome and quality of life in the treatment groups between treatment arms will be evaluated using a linear regression model with repeated measures.
- Two-year implant survival will be evaluated according to the Kaplan-Meier [6] method and compared in the treatment arms with log-rank test for the events.
  -
    - ◦ Periprosthetic joint infections as defined for the primary endpoint
    - ◦ Septic revisions
    - ◦ All-cause revisions
- Radiological outcome in the treatment groups for the endpoints
  - ◦ Implant migration
  - ▪ Occurrence of hip stem migration > 2 mm by using a logistic regression model with repeated measures
  - ▪ Radiological signs of loosening
  - ▪ Mean migration in patients with relevant migration (> 2 mm) by using a linear regression model with repeated measures
  - ◦ Radiologic changes in the fixation area
  - ▪ Occurrence of any radiolucency by using a logistic regression model with repeated measures
- Safety outcomes of adverse events, device failure, and component revision between treatment arms will be evaluated by using a logistic regression.

Silver ion concentrations in peripheral blood and drainage fluid will be descriptively analyzed.

Results of statistical tests are evaluated using a *p*-value. The significance level is set to 5% two-sided and 2.5% one-sided, respectively. Two-sided 95% confidence intervals may be given in addition.

SAS VIYA 4 statistical software (SAS Institute Inc., Cary, NC, USA) or an adequately suited alternative will be used to generate the analysis datasets and to perform statistical calculations. The R package rpact from CRAN is used to calculate the combination of tests for primary analysis.

### Interim analyses {21b}

Three interim analyses are planned:

- Interim analysis I will be conducted after the pilot phase for safety assessment of the 6 weeks’ results of part A subjects. All AEs of the safety population will be summarized for the analysis. Where applicable, descriptive statistics may be used based on AE categories. No analysis of the primary endpoint, secondary performance, or other variables will be performed.
- Interim analysis II is planned for the purpose of sample size re-estimation. The sample size re-estimation will be performed if either the first 134 patients have passed their 12-month follow-up time point or when more than 90% of the subjects of the initially calculated sample size (242 or more subjects) have been treated, whatever happens first. The interim analysis is performed based on unblinded data, because the effect size and the events in the control group cannot be reliably predicted. No confirmatory test is carried out during interim analysis II (i.e., no early efficacy or futility stop).
- The interim analysis III includes analyses of primary and secondary efficacy endpoints, based on the intention-to-treat population. The primary endpoint analysis will be conducted as soon as the 12-month follow-up period of the stage 2 patients is completed.

### Methods for additional analyses (e.g., subgroup analyses) {20b}

Subgroup analyses will explore outcomes stratified by patient factors such as surgical treatment (one-stage vs. two-stage revision), infection severity, and baseline functional status. Adjusted analyses may be conducted using multivariable regression models to account for potential confounders (e.g., age, comorbidities, and ASA physical status).

### Methods in analysis to handle protocol nonadherence and any statistical methods to handle missing data {20c}

The primary analysis will follow the intent-to-treat (ITT) principle, including all eligible randomized participants in the groups they were assigned. A per-protocol (PP) analysis will be conducted as a sensitivity analysis, excluding participants with significant protocol deviations.

Statistical methods (e.g., multiple imputation) for handling missing values are not used. In the case of a partial date such as adverse event onset date or date of death, the unknown portion of the date of the event will be imputed. If the month and year are known, the 15th of the month will be used for analysis. If only the year is known, the event will be analyzed as if it occurred on June 30 of the known year. In the rare case that the date is fully unknown, the date will be imputed as the operation date.

Dropout reasons will be carefully documented to assess potential biases.

### Plans to give access to the full protocol, participant-level data, and statistical code {31c}

Following study completion and publication, de-identified participant-level data, full trial protocol, and statistical analysis code will be made available upon reasonable request to qualified researchers, in accordance with data sharing policies and regulations.

## Oversight and monitoring

### Composition of the coordinating center and trial steering committee {5d}

The coordinating center will oversee day-to-day study operations, data management, and communications with sites. An independent data monitoring committee will review safety data and advise on trial continuation.

### Composition of the data monitoring committee and its role and reporting structure {21a}

An independent data monitoring committee (DMC) will be established to monitor patient safety as the study progresses. The committee consists of acknowledged experts in study-related fields and an independent biostatistician, not otherwise associated with the trial, and acts in a senior advisory capacity on policy matters. This committee watches over the ethics of conducting the study in accordance with the Declaration of Helsinki. If a serious concern with the safety of the patients in the trial were to arise, the data and safety monitoring committee may recommend early termination of the study. All final decisions regarding study modifications or termination will be made by the sponsor. The sample size re-estimation will be performed by the independent DMC biostatistician. The DMC will provide a recommendation to increase the sample size or to continue the study with the originally planned sample size, taking into account the risk–benefit profile of the investigational device. The decision to increase the sample size will be made by the sponsor based on the recommendation of the DMC. Detailed information, such as the DMC member selection, composition, duties, procedures, and deliberation rules, is detailed and documented in the DMC Charter.

### Frequency and plans for auditing trial conduct {23}

Trial conduct may be audited by an independent quality assurance team not involved in the study’s execution. Audits will review adherence to the protocol, regulatory compliance, and data integrity, with findings reported to the sponsor and ethics committees as appropriate.

### Plans for communicating important protocol amendments to relevant parties (e.g., trial participants and ethical committees) {25}

Significant protocol amendments will be communicated in a timely manner to ethics committees, regulatory authorities, participating sites, and, where applicable, to study participants. All amendments will be implemented only after received approvals.

### Adverse event reporting and harms {22}

All AEs and device deficiencies mentioned by the patient or observed by an investigator will be reported together with the causality via the eCRF. SAEs will be reported to authorities according to the applicable regulations.

### Dissemination plans {31a}

Both positive and negative results will be reported in international peer-reviewed journals.

## Discussion

To the authors’ best knowledge, this is the first randomized controlled superiority trial evaluating the outcome of silver-coated versus non-silver-coated prostheses for the treatment of hip PJI. The results of this clinical investigation will provide valuable insights into the potential of IP-coated hip implants to improve clinical outcomes in PJI. Given the significant burden of PJI and the associated healthcare costs, the study’s findings have the potential to influence clinical decision-making and implant selection for revision hip procedures in the future. If the IP-coated implants demonstrate a significant reduction in infection rates, they could become a valuable addition to the standard armamentarium for infection-prone patients.

For market approval of an antibacterial coating, demonstrating safety alone is insufficient; a clear clinical benefit with improvement of clinical outcome in PJI treatment must be shown through comparative clinical data. The current study with its prospective randomized controlled design meets these requirements by directly comparing the IP-coated implants against non-IP-coated comparator devices. Positive findings from this investigation would support regulatory approval by providing the required clinical evidence that the antimicrobial coating reduces PJI risk in revision hip arthroplasty compared to the current standard of care.

One of the study’s strengths is its rigorous superiority design, which includes well-defined eligibility criteria, ensuring the inclusion of patients at high risk for reinfection while maintaining generalizability in hip PJI treatment. Additionally, the use of randomization via the electronic database, allocation concealment, and blinded radiological analysis minimizes bias and enhances the reliability of results. The study also benefits from its sample size calculation based on real-world data from the German Arthroplasty Registry (EPRD), increasing the likelihood that findings will be applicable to routine clinical practice.

However, several limitations must be acknowledged. First, the single-blinded design means that surgeons are aware of the implant type being used, which could introduce performance bias. While the use of the objective EBJIS criteria mitigates this risk, subtle differences in surgical handling or postoperative management could theoretically influence outcomes. Second, the study’s follow-up period of 12 months is sufficient to capture early postoperative infections but may not fully assess long-term implant performance and infection prevention. Longer-term follow-up studies will be necessary to confirm sustained benefits of the antimicrobial coating beyond 2 years postoperatively. Another potential limitation is the variability in surgical techniques and institutional infection control protocols across different study sites, which could influence outcomes. Additionally, the study is conducted in different countries with different microbial spectra and resistances, which could further impact the results. While the study attempts to standardize surgical and postoperative protocols, inherent differences between surgeons and institutions remain a potential source of heterogeneity.

If the study confirms a significant reduction in PJI rates with IP-coated implants, future research should focus on long-term outcomes, including implant survival, functional performance, and patient-reported quality of life. Additional studies should also evaluate the cost-effectiveness of this technology, considering the potential reduction in infection-related hospitalizations, reoperations, and antibiotic use. Moreover, exploring the applicability of this infection-preventive strategy to other orthopedic implants, such as knee or shoulder revisions, could further broaden its clinical impact.

Conversely, if the study fails to demonstrate a significant reduction in infection rates, further investigations will be needed to refine the antimicrobial coating technology or explore alternative strategies for infection prevention. Understanding the reasons behind potential treatment failure—whether related to coating durability, bacterial resistance, or host factors—will be crucial in guiding future innovation in orthopedic biomaterials.

In conclusion, the current study tries to fill the evidence gap for silver-coated implants to reduce re-infection rates in the treatment of hip PJI. To the authors’ best knowledge, this is the first randomized controlled superiority trial evaluating the outcome of silver-coated versus non-silver-coated implants. Results of this trial will most likely have a significant impact on the use of silver-coated implants for PJI treatment in the future.

## Trial status

The first patient was treated on 25 June 2025, and last patient’s last visit is expected for Q3 2028. Protocol version is 5.0, 21 March 2025.

## Data Availability

The sponsor plans to provide access to the study protocol, anonymized participant-level data, and statistical code upon request to researchers after study completion. These requests will be evaluated based on scientific merit and compliance with applicable regulations, ensuring participant confidentiality. Access procedures will be detailed in the final publication.

## Abbreviations

ADE: Adverse device effect
AE: Adverse event
ALT: Alanine transaminase
ASA: American Society of Anesthesiologists
AST: Aspartate transaminase
CE: Conformité Européenne (European Conformity)
CIP: Clinical Investigation Plan
CRP: C-reactive protein
DAIR: Debridement, antibiotics, and implant retention
DSMB: Data and Safety Monitoring Board
EBJIS: European Bone and Joint Infection Society
eCRF: Electronic case report form
eGFR: Estimated glomerular filtration rate
EPRD: German Arthroplasty Registry (Endoprothesenregister Deutschland)
EQ-5D: EuroQol 5-Dimension questionnaire
GCP: Good Clinical Practice
GGT: Gamma-glutamyl transferase
HHS: Harris Hip Score
IP: Infection prevention
ITT: Intention to treat
MAR: Missing at random
PJI: Periprosthetic joint infection
SADE: Serious adverse device effect
SAE: Serious adverse event
SOP: Standard operating procedure
THA: Total hip arthroplasty

## References

[1] EPRD Jahresbericht 2023. DE: EPRD Endoprothesenregister Deutschland; 2023 [zitiert 29. Januar 2025]. 175 S. Verfügbar unter: 10.36186/reporteprd082023

[2] Reinhard J, Lang S, Walter N, Schindler M, Bärtl S, Szymski D, u. a. In-hospital mortality of patients with periprosthetic joint infection: demographic, comorbidity, and complication profiles of 52,286 patients. Bone Jt Open. 26. April 2024 [zitiert 29. Januar 2025];5(4):367–73. Verfügbar unter: https://boneandjoint.org.uk/doi/10.1302/2633-1462.54.BJO-2023-0162.R1 10.1302/2633-1462.54.BJO-2023-0162.R1 PMC11045279 38663864

[3] Szymski D, Walter N, Hierl K, Rupp M, Alt V. Direct hospital costs per case of periprosthetic hip and knee joint infections in Europe — a systematic review. J Arthroplasty. Juli 2024 [zitiert 29. Januar 2025];39(7):1876–81. Verfügbar unter: https://linkinghub.elsevier.com/retrieve/pii/S0883540324000329 10.1016/j.arth.2024.01.032 38266688

[4] Shichman I, Sobba W, Beaton G, Polisetty T, Nguyen HB, Dipane MV, u.a. The effect of prosthetic joint infection on work status and quality of life: a multicenter, international study. J Arthroplast. Dezember 2023 [zitiert 3. Februar 2025];38(12):2685–2690.e1. Verfügbar unter: https://linkinghub.elsevier.com/retrieve/pii/S088354032300654X 10.1016/j.arth.2023.06.015 37353111

[5] Wildeman P, Rolfson O, Söderquist B, Wretenberg P, Lindgren V. What are the long-term outcomes of mortality, quality of life, and hip function after prosthetic joint infection of the hip? A 10-year follow-up from Sweden. Clin Orthop Relat Res. Oktober 2021 [zitiert 3. Februar 2025];479(10):2203–13. Verfügbar unter: https://journals.lww.com/10.1097/CORR.0000000000001838 10.1097/CORR.0000000000001838 PMC8445574 34061486

[6] Fabritius M, Al-Munajjed AA, Freytag C, Jülke H, Zehe M, Lemarchand T, u.a. Antimicrobial silver multilayer coating for prevention of bacterial colonization of orthopedic implants. Materials. 20. März 2020 [zitiert 28. Januar 2025];13(6):1415. Verfügbar unter: https://www.mdpi.com/1996-1944/13/6/1415 10.3390/ma13061415 PMC7143109 32245004

[7] Khalilpour P, Lampe K, Wagener M, Stigler B, Heiss C, Ullrich MS, u.a. Ag/SiO_x_ C_y_ plasma polymer coating for antimicrobial protection of fracture fixation devices. J Biomed Mater Res. Juli 2010 [zitiert 28. Januar 2025];94B(1):196–202. Verfügbar unter: https://onlinelibrary.wiley.com/doi/10.1002/jbm.b.31641 10.1002/jbm.b.31641 20524195

[8] Alt V. Antimicrobial coated implants in trauma and orthopaedics–a clinical review and risk-benefit analysis. Injury. März 2017 [zitiert 28. Januar 2025];48(3):599–607. Verfügbar unter: https://linkinghub.elsevier.com/retrieve/pii/S0020138316308178 10.1016/j.injury.2016.12.011 28088378

[9] Alt V, Heiss C, Rupp M. Treatment of a recurrent periprosthetic joint infection with an intramedullary knee arthrodesis system with low-amount metallic silver coating. J Bone Joint Infect. 20. April 2019 [zitiert 28. Januar 2025];4(3):111–4. Verfügbar unter: https://jbji.copernicus.org/articles/4/111/2019/10.7150/jbji.34484 PMC6536804 31192109

[10] Alt V, Rupp M, Lemberger K, Bechert T, Konradt T, Steinrücke P, u.a. Safety assessment of microsilver-loaded poly(methyl methacrylate) (PMMA) cement spacers in patients with prosthetic hip infections: results of a prospective cohort study. Bone & Joint Research. August 2019 [zitiert 28. Januar 2025];8(8):387–96. Verfügbar unter: https://online.boneandjoint.org.uk/doi/10.1302/2046-3758.88.BJR-2018-0270.R1 10.1302/2046-3758.88.BJR-2018-0270.R1 PMC6719530 31537996

[11] McNally M, Sousa R, Wouthuyzen-Bakker M, Chen AF, Soriano A, Vogely HC, u.a. The EBJIS definition of periprosthetic joint infection: a practical guide for clinicians. The Bone & Joint J. 1. Januar 2021 [zitiert 28. Januar 2025];103-B(1):18–25. Verfügbar unter: https://online.boneandjoint.org.uk/doi/10.1302/0301-620X.103B1.BJJ-2020-1381.R1

[12] Pocket Guide to Diagnosis & Treatment of Periprosthetic Joint Infection (PJI). Pro Implant. [zitiert 28. Januar 2025]. Verfügbar unter: https://pro-implant.org/product/pocket-guide-to-diagnosis-treatment-of-periprosthetic-joint-infection-pji/

[13] Doyle DJ, Hendrix JM, Garmon EH. American Society of Anesthesiologists Classification. In: StatPearls. Treasure Island (FL): StatPearls Publishing; 2025 [zitiert 3. Februar 2025]. Verfügbar unter: http://www.ncbi.nlm.nih.gov/books/NBK441940/

[14] Ramadanov N, Voss M, Hable R, Hakam HT, Prill R, Salzmann M, u.a. Postoperative Harris Hip Score versus Harris Hip Score difference in hip replacement: what to report? Orthopaedic Surg. Januar 2025 [zitiert 28. Januar 2025];17(1):3–21. Verfügbar unter: https://onlinelibrary.wiley.com/doi/10.1111/os.14272 10.1111/os.14272 PMC11735366 39434235

[15] Devlin N, Pickard S, Busschbach J. The development of the EQ-5D-5L and its value sets. In: Devlin N, Roudijk B, Ludwig K, Herausgeber. Value Sets for EQ-5D-5L. Cham: Springer International Publishing; 2022 [zitiert 28. Januar 2025]. S. 1–12. Verfügbar unter: https://link.springer.com/10.1007/978-3-030-89289-0_1 36810043

[16] Fabritius M, Al-Munajjed AA, Freytag C, Jülke H, Zehe M, Lemarchand T, u.a. Antimicrobial silver multilayer coating for prevention of bacterial colonization of orthopedic implants. Materials. 20. März 2020 [zitiert 27. Januar 2026];13(6):1415. Verfügbar unter: https://www.mdpi.com/1996-1944/13/6/1415 10.3390/ma13061415 PMC7143109 32245004

[17] Pagès G, Hammer M, Grand JG, Irubetagoyena I. Long-term outcome of tibial plateau leveling osteotomy using an antimicrobial silver-based coated plate in dogs. Abu-Seida AM, Herausgeber. PLoS ONE. 12. August 2022 [zitiert 27. Januar 2026];17(8):e0272555. Verfügbar unter: https://dx.plos.org/10.1371/journal.pone.0272555 10.1371/journal.pone.0272555 PMC9374240 35960740

[18] Wassmer G, Brannath W. Group Sequential and Confirmatory Adaptive Designs in Clinical Trials. Cham: Springer Nature Switzerland; 2025 [zitiert 27. Januar 2026]. (Springer Series in Pharmaceutical Statistics). Verfügbar unter: https://link.springer.com/10.1007/978-3-031-89669-9

